# Veterinary Progress in Michigan1Read at the Thirty-seventh Annual Meeting of the American Veterinary Medical Association, Detroit, September, 1900.

**Published:** 1900-11

**Authors:** William Jopling

**Affiliations:** Owosso, Mich.


### VETERINARY PROGRESS IN MICHIGAN.[1]

By William Jopling, V.S.,
OWOSSO, MICH.

I have had occasion to wonder many times in the past month what you gentlemen are expecting to hear from me under the title of my paper, "Veterinary Progress in Michigan."

What I should say, or should not say, has caused me no little anxiety. This is my first appearance as a contributor to the programme of the A. V. M. A., and in making my debut, I must acknowledge a feeling of temerity. If I had not felt it an imperative duty I would not be standing before you now. I have been too long intimately associated with our State Association not to be aware of the difficulty in making up a programme. Various reasons might be given explanatory of this condition of affairs, but it is nevertheless a fact that the majority of practitioners are loath to commit their thoughts and experiences to papers for the benefit and entertainment of their colleagues. This at least has been my experience with Michigan veterinarians. With this idea in mind I have taken upon myself the preparation of this paper, in the sense that it is a duty I owe the members of the A. V. M. A., who have honored our State of Michigan by their presence within our borders at this time. There are also a few matters explanatory of conditions existing among the veterinary profession of this State which I feel called upon to lay before you.

The title of my paper was not of my own selecting, but was suggested to me by request. Living in a quiet country town within whose precincts tuberculosis, glanders, rabies, swine-plague, anthrax, and kindred diseases are practically unknown, and which furnish

[1] Read at the Thirty-seventh Annual Meeting of the American Veterinary Medical Association, Detroit, September, 1900.

popular themes for contributors to journals and such meetings as this, I felt were not within my province, as not having personal experience with them, I could produce nothing new and perhaps subject myself to the charge of plagiarism should I attempt it. In fact, the country in my vicinity appears to be in a very healthy condition, so much so that the much-mooted question of milk- and meat-inspection would not furnish me a theme. So leaving those questions to abler minds and those more favorably situated to write upon and discuss them, and asking your forbearance for this lengthy introduction, I will return to my text.

The time is too limited ; the conditions unfavorable, and their usefulness at this time too doubtful to furnish any statistics on veterinary matters in this State. Looking back for a period of twenty years, I am informed that then there were only two graduates in the State. Butchers, or men travelling with stallions, hostlers, etc., were applied to when assistance was needed. Since that time there has been a steady influx of college graduates till I think, I may say, there are over two hundred in the State at the present time. I am of the opinion that the law of the survival of the fittest will apply in this case, and that the charlatans and pretenders are gradually giving place to educated men, and now they are as few as formerly they were plentiful, and without the aid of legislative enactment.

Like in other States and Provinces, the veterinarians formed themselves into an association. This was some seventeen years ago. The organization has passed through the various periods of energetic activity at times, followed by periods of lethargy, a condition which I suppose all such societies experience. We have now seventy members enrolled, and are very harmonious and prosperous—speaking in a general way.

Being a strong advocate and lover of association work myself, the apparent absence of that feeling in many veterinarians is somewhat mysterious. Graduating as many of us did fifteen or twenty years ago, with the absence of chances of post-graduate work, such as the medical profession enjoy, how can we better keep abreast of the times than through and by the aid of these associations, where the interchange of thought and experiences must certainly benefit us individually and collectively? I feel somewhat ashamed of my professional brethren to find only four names on the roll of this Association. Why is this so? Reasons have been given, such as its being too expensive and too far to go to attend the sessions, etc. I became a member of this Association several years ago, and this one and the meeting in Chicago are the only ones I have had an opportunity to attend, for reasons over which I had no control, but I receive after each annual meeting the *Proceedings* thereof, and feel amply repaid.

When I ponder over these questions and consider the why and wherefore, I am compelled to think that were the matters referred to my friend Hoskins, of the JOURNAL, for explanation he would attribute it to want of higher veterinary education. It also surprises me when I learn of the comparatively few who are subscribers to our veterinary journals. This does not seem right.

Speaking of journals, I will presuppose that all members of this Association read the JOURNAL OF COMPARATIVE MEDICINE AND VETERINARY ARCHIVES. This granted, you will all be aware of the conspicuous place Michigan has taken in its pages during the past year. You will be led thereby to think our State is in a bad plight. Whether correct or not, I look upon this JOURNAL as, in a sense, the official organ of the A. V. M. A. I assume this from the very close relations its editor has sustained toward it this many years. This being the case, I beg your indulgence while we look into the matter a little.

According to the JOURNAL, Detroit is and has been the storm-centre of professional disturbances. That the cause of higher veterinary education has received its death-knell for a time in this State ; that her schools are closing their doors or demoralized in a supreme effort to gain personal advantage and supremacy in active competition for students by an attractive short term ; that our State Association has not been powerful enough to sustain her high-grade schools, or to mould their legislation in the interests of higher veterinary education now or progressively ; that we have not been strong enough in State affairs to even obtain the appointment on this body of our deliberate selections ; that the time is auspicious for a meeting in this State, for the profession is rent asunder with internal strife for leadership ; that the chief veterinary official of the State is openly charged with incompetency in the daily papers throughout the Commonwealth ; that the lack of support by the profession and the State has closed the doors of a school that endeavored to maintain the standard of the A. V. M. A., and sent from the State one of the ablest veterinarians she possessed ; that a rival school, the outcome of the high course at Detroit, established by one who was unable to maintain the school at the latter point and which now maintains a curriculum that makes her graduates ineligible to membership in the A. V. M. A., and which is practically a one-man school; that the State Association is not a unit on any of the leading questions of the day for the welfare of the profession; that the time is propitious; that the hour is one of need for the presence and assistance of some strong power to aid in the solution of these trying conditions.

The JOURNAL closes with a hope that the Convention of the A. V. M. A. will prove a panacea for all these ills.

I find it humiliating to admit that there are grains of truth in some of those assertions; but I assure you, gentlemen, that many of them are erroneous and misleading and work an injustice to many of us.

It will not be necessary for us in Michigan to climb a tree to see where the JOURNAL gets its information, and I cannot help but think that if the editor would use more discretion in the use of the waste-basket there might be more harmony and the cause of veterinary education be enhanced in the State. It may be that it has received its death-knell; but may we not hope that all this disturbance, strife, and agitation by awakening renewed interest may be a means toward an end that will produce a new era and restore or bring us to a higher basis.

I dislike these disturbances very much, but hope good may come in the near future out of the evil of to-day.

I feel like denying that the State Association had anything to do with closing the Detroit school. Not being endowed or supported by the State, it simply went out of business the same as might have occurred in any other State where a college was not needed. I am also of the opinion that our ablest veterinarian did not leave the State on that account.

The institution at Grand Rapids was evidently started by one who, unable to gain admission or graduate from schools then in existence, built one for himself that he could control and graduate from.

The fact that he or none of the staff (unless there may be those who graduated from the Ontario College prior to 1893) are eligible to membership in this Association will help you to draw your own conclusions as to the chances of its promoting the cause of higher veterinary education.

This school is a blot on the civilization of our State, otherwise famed for its educational institutions. As I have said of the Detroit school, there was seemingly no need of this one, and if there was it had no excuse for not commencing with a three-year course, except from a financial point of view. I have had occasion to pay my respects to its dean through the JOURNAL. He has returned the compliment, and you will know that I am plain Mr. Jopling (liveryman), if you please. I acknowledge the corn, gentlemen. Graduates from the Ontario College do not aspire to the title " Doctor," but from the indiscriminate use made of the term the honor is doubtful in having it prefixed to your name.

But I can claim the honor of being a member of this Association, an honor which Professor Conkey is not likely ever to attain.

It is very distasteful to me to write upon such matters as this, but conditions seem to demand that I should, and I hope you will bear with me. This person divides the State into two elements —the American and the foreign element. The latter, or the graduates of the Ontario College, I venture to say constitute nine-tenths of the profession in the State. The former is made up of Professor Conkey, the (*Hon. W. W. Thorburn, V.S., D.V.S.*) Secretary of the State Veterinary Board, and evidently a traitor to our Association and others.

Like Americans usually do, they won the battle, but unlike Americans do, they did it treacherously, and by that means beat the somewhat unsophisticated foreign element at every turn of the hat in a political and legislative way. So that perhaps, as the JOURNAL says, " Better that we have no law at all."

As I have stated, there was no good reason why the Grand Rapids institution should not have started its career with a three-year course.

Neither was there sufficient reason why the Ontario College should not have extended its curriculum to three years—further than that, having been thoroughly established in the old rut for so many years, it was more difficult to adopt the new idea. I am a sympathizer with Dr. Hoskins in his efforts to secure higher veterinary education. The Ontario school is my alma mater, but I cannot help but feel like censuring the management of that institution for not extending its course to three years and meeting the requirements of the A. V. M. A., and I feel safe in saying that the majority of her graduates feel likewise.

It works an injustice to her alumni since 1893, and we have graduates in this State debarred from this Association who otherwise are entitled to membership. Of course, it may be said that it is a prospective student's privilege to choose his college, and, being aware of the conditions, abide thereby.

I am convinced that they make an error in the commencement of their career by so doing. Three years is a short enough time in which to receive a finished education such as is required the present day. I think you will concede that the graduates of the Ontario College, as a rule, are a good all-around practical class of men who meet the requirements as ordinarily met within a satisfactory manner.

I am satisfied in my own mind that one of the weakest points in connection with the Ontario College is the low standard of its matriculation examination.

Let a graduate of one of our high schools enter that institution to-day, and if he does his part can emerge at the end of the course with an education which will be a credit to himself and a benefit to the world.

But when a student enters who, so to speak, can with difficulty write his name, what are we to expect when you talk of higher veterinary education? This may appear to you like plain speech, but the conditions seem to demand plain talk.

With a few remarks upon legislation, I will close. I have never been enthusiastic on this question; I have often looked upon it as the bane of our Association, always consuming much time in discussion and depleting our treasury of funds without seemingly making any progress unless the constant agitation kept up year after year will do good by keeping the question before the public eye.

It seems to me a sad commentary on our civilization that every act of our lives, nearly, has to be regulated by statutes. To sum up, it seems to be a case of choosing between two evils—law or no law. Veterinarians in Michigan have chosen the former as the lesser evil, and I am authorized by the chairman of our Committee on Legislation to say to you that our veterinary bill was the strongest they could get. That it passed the House of Representatives with a provision that all schools and colleges to be recognized were only those that were recognized by the A. V. M. A., but that it was held up in the Senate by the Dean of the Grand Rapids School, which is not recognized by said Association, and we had to amend our bill in order to get it recommended by the Senate committee. However, we expect to have said bill amended at the next session of the Legislature—said amendment taking effect, say the 1st of January, 1901—providing that colleges to be recognized shall be up to the standard recognized by the A. V. M A.; also cutting out the privilege of an examination by the board, and that we propose to strengthen the bill in other ways of minor importance.

I am also informed that there is a bill to be introduced at the next session of the Legislature, by Dr. Baker, Secretary of the State Board of Health, which is of interest to local veterinarians. This bill is to regulate the sale of milk and the examination of dairies and dairy cows in the incorporated villages and cities of Michigan. It is the purpose of our Committee on Legislation to have a clause in said bill providing that all inspectors and examinations shall be made by registered veterinarians, and that they shall work with their local boards of health.

